# Sediment deposition from eroding peatlands alters headwater invertebrate biodiversity

**DOI:** 10.1111/gcb.14516

**Published:** 2018-12-01

**Authors:** Lee E. Brown, Katie L. Aspray, Mark E. Ledger, Chris Mainstone, Sheila M. Palmer, Martin Wilkes, Joseph Holden

**Affiliations:** ^1^ Water@Leeds, School of Geography University of Leeds Leeds UK; ^2^ School of Geography, Earth and Environmental Science University of Birmingham Birmingham UK; ^3^ Natural England Peterborough UK; ^4^ Centre for Agroecology, Water and Resilience Coventry University Coventry UK

**Keywords:** erosion, functional diversity, headwater, macroinvertebrate, peat, river, stream, traits

## Abstract

Land use and climate change are driving widespread modifications to the biodiverse and functionally unique headwaters of rivers. In temperate and boreal regions, many headwaters drain peatlands where land management and climate change can cause significant soil erosion and peat deposition in rivers. However, effects of peat deposition in river ecosystems remain poorly understood. We provide two lines of evidence—derived from sediment deposition gradients in experimental mesocosms (0–7.5 g/m^2^) and headwaters (0.82–9.67 g/m^2^)—for the adverse impact of peat deposition on invertebrate community biodiversity. We found a consistent negative effect of sediment deposition across both the experiment and survey; at the community level, decreases in density (1956 to 56 individuals per m^2^ in headwaters; mean 823 ± 129 (*SE*) to 288 ± 115 individuals per m^2^ in mesocosms) and richness (mean 12 ± 1 to 6 ± 2 taxa in mesocosms) were observed. Sedimentation increased beta diversity amongst experimental replicates and headwaters, reflecting increasing stochasticity amongst tolerant groups in sedimented habitats. With increasing sedimentation, the density of the most common species, *Leuctra inermis*, declined from 290 ± 60 to 70 ± 30 individuals/m^2^ on average in mesocosms and >800 individuals/m^2^ to 0 in the field survey. Traits analysis of mesocosm assemblages suggested biodiversity loss was driven by decreasing abundance of invertebrates with trait combinations sensitive to sedimentation (longer life cycles, active aquatic dispersal of larvae, fixed aquatic eggs, shredding feeding habit). Functional diversity metrics reinforced the idea of more stochastic community assembly under higher sedimentation rates. While mesocosm assemblages showed some compositional differences to surveyed headwaters, ecological responses were consistent across these spatial scales. Our results suggest short‐term, small‐scale stressor experiments can inform understanding of “real‐world” peatland river ecosystems. As climate change and land‐use change are expected to enhance peatland erosion, significant alterations to invertebrate biodiversity can be expected where these eroded soils are deposited in rivers.

## Graphical abstract

Land use and climate change are driving widespread modifications to the biodiverse and functionally unique headwaters of rivers. In temperate and boreal regions, many headwaters drain peatlands where land management and climate change can cause significant soil erosion and peat deposition in rivers. We provide two lines of evidence—derived from sediment deposition gradients in experimental mesocosms and headwaters—for the adverse impact of peat deposition on invertebrate community biodiversity.

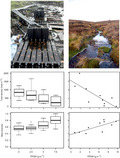

## 1 INTRODUCTION

The headwaters of river systems make a major contribution to global aquatic biodiversity. Headwaters constitute a majority of the total length of rivers, but these heterogeneous, dynamic environments are geographically isolated such that dispersal limitation maintains high beta diversity across the river network (Brown et al., 2018; Finn, Bonada, Múrria, & Hughes, 2011; Tonkin, Heino, & Altermatt, 2018). Headwaters maintain the ecological functioning of whole river networks because biological assemblages in downstream habitats depend on headwater streams for organic matter supply and biota recruitment (Wipfli, Richardson, & Naiman, 2007). Despite their importance to river health, headwaters remain underrepresented in biological monitoring programmes in many regions of the world (Dunbar et al., 2010), leading to knowledge gaps for effective river biodiversity conservation and ecosystem services management. As a consequence, stressors associated with headwater catchment and river channel alterations due to land management activities or global change can have undetected, but often disproportionately large, effects on aquatic biodiversity (Harding, Benfield, Bolstad, Helfman, & Jones, 1998; Piggott, Townsend, & Matthaei, 2015).

Northern temperate and boreal region peatlands account for an estimated 80%–90% of the 4.23 M km^2^ of peat cover on Earth (Xu, Morris, Liu, & Holden, 2018). The headwaters of many major river systems originate from these peatlands, where a water surplus leads to slow rates of decomposition and the build‐up of low‐density organic soil cover (Charman, 2002). Peatlands naturally release organic matter to river systems as both dissolved and particulate loads, but these effects can be amplified as a direct consequence of land management change or climate change (Li, Irvine, Holden, & Mu, 2017b). Using an ensemble of climate change predictions to 2100, Li, Holden, Irvine, and Mu (2017a) parameterized a peat erosion model to show that temperature increase will be a key driver of enhanced blanket peat erosion across the Northern Hemisphere. Average annual sediment yields were predicted to increase by around 14%. High spatial variability in future erosion increases was forecast with some warmer and lower latitude regions more at risk. However, even within the British Isles Li, Holden, and Irvine (2016) predicted a doubling of sediment yield for some sites by 2100. Global climate change further threatens high‐latitude permafrost peatlands, due to thawing, degradation and slumping (Kokelj et al., 2013; Swindles et al., 2015), which may release large quantities of carbon in river systems. Severe air pollution has led to enhanced physical erosion of peatlands due to vegetation loss in some regions (Holden et al., 2007; Yeloff, Labadz, Hunt, Higgitt, & Foster, 2005). Northern peatlands have historically been subject to intensive drainage to lower the water table in an attempt to make the land more suitable for animals, arable agriculture, forestry and/or gun‐sports, but in places cause up to 200‐fold increases in river sediment loads (Ahtiainen & Huttunen, 1999; Prévost, Plamondon, & Belleau, 1999; Ramchunder, Brown, & Holden, 2009, 2012). In some areas, this enhanced erosion has been due to decay of the peatland through subsurface evacuation of sediment from large cavities (peat pipes) (Holden et al., 2012), a feature that appears to be exacerbated by installation of drainage ditches (Holden, 2006). In other areas, peat is extracted for use as fuel or in horticulture (Waddington, Plach, Cagampan, Lucchese, & Strack, 2009), and vegetation is removed to prevent wildfire, to promote grazing or to enhance game bird density for gun‐sports leading to the exposure and erosion of soils (Brown et al., 2015).

Disturbed and exposed organic soils are vulnerable to erosion due to their low density, which ultimately leads to enhanced delivery of particulate organic matter to rivers. In peatlands, this effect is increased by the dominance of saturation‐excess overland flow processes and movement by wind (Li, Holden, & Grayson, 2018b). Particulates have been shown to constitute up to 75% of the organic load of some temperate‐zone blanket peatland rivers (Evans & Warburton, 2007), while permafrost–slump sediment inputs can dominate particulate organic fluxes in high Arctic rivers (Lamoureux & Lafrenière, 2014). Severe erosion of organic soils presents the potential for major changes to the biodiversity of receiving headwaters via modifications to river habitat, smothering of the benthos, and modification of functional processes such as primary production which provide energy to aquatic food webs (Aspray, Holden, Ledger, Mainstone, & Brown, 2017; Chin, Lento, Culp, Lacelle, & Kokelj, 2016). However, knowledge of aquatic biodiversity and trait responses to organic soil deposition in rivers is lacking when compared to the effects of inorganic sand and silt (Jones et al., 2012; Larsen & Ormerod, 2010b; Mustonen et al., 2016). The use of traits to develop mechanistic understanding of invertebrate community responses to fine sedimentation is growing (Descloux, Datry, & Usseglio‐Polatera, 2014; Murphy et al., 2017), but it remains unclear whether trait responses to organic sediments are the same as inorganic sediments. These knowledge gaps prevent peatland managers from understanding the significance of soil erosion in terms of effects on biodiversity responses in nearby aquatic systems. There is a clear need to (a) generate experimental evidence to understand the direct impacts of organic sediment deposition on invertebrate biodiversity because much of our existing knowledge is from correlative field surveys in which sediment gradients may be confounded with other stressors, (b) develop an understanding of the underlying mechanisms driving any biodiversity responses via trait‐based analyses and (c) demonstrate that where sedimentation is a significant stressor in peatland headwater river ecosystems, invertebrate biodiversity responds in a similar manner to controlled experimental systems so that managers can be confident that mitigating sediment pressures will produce beneficial biodiversity gains.

The impact of organic sedimentation on aquatic ecosystems can vary depending on loading rates. For example, light organic sedimentation (such as might be encountered in hydrologically intact peatlands) can increase production of river ecosystems, providing food for detritivores (Peeters, Brugmans, Beijer, & Franken, 2006) and enhancing phosphorus retention (Aldridge, Brookes, & Ganf, 2009). However, these effects may be reversed when loading rates are increased. For instance, heavy sedimentation is thought to be a key driver of biodiversity loss in peatland rivers where fire is used to remove catchment vegetation (Brown, Johnston, Palmer, Aspray, & Holden, 2013; Ramchunder, Brown, & Holden, 2012, 2013). However, these studies were correlational surveys, lacking the control for confounding variables that can be achieved with experimental manipulations. Detailed studies of ecosystem responses where peatlands have eroded more severely are required to understand better the effects of highly amplified particulate organic matter supply to freshwater systems (Thienpont et al., 2013). Improving our understanding of organic sediment impacts on sensitive headwaters is vital to inform land management in the face of predicted future environmental change, and for guiding restoration efforts that seek to mitigate soil erosion in currently impaired systems (Li, Holden, et al., 2017a).

In this study, we developed new insights into the effects of peatland erosion and sedimentation on river invertebrate communities via comparative evaluations of aquatic biodiversity responses in: (a) a fully controlled and replicated riverside mesocosm experiment examining the impacts of benthic organic sedimentation on aquatic invertebrates, and (b) surveys of peatland rivers across the Pennine region of northern England with different levels of fine organic matter deposits on the bed. We focused on both taxonomic and trait‐based measures of biodiversity, with the latter adopted as a potential means of evaluating the mechanistic basis of any taxonomic responses. Experimental studies allowed an assessment of the direct effects of sedimentation on weekly–monthly timescales while controlling for confounding effects that are commonly encountered in field surveys. Complementary field surveys can reveal the products of integrated stressor effects over longer time periods. This combination of approaches allows us to establish the potential benefits of short‐term, small‐scale stressor experiments in informing understanding of “real‐world” peatland headwater ecosystems given the potential importance of scale‐specific effects seen in other studies (Larsen, Vaughan, & Ormerod, 2009). The study aimed to test three hypotheses: (H_1_) increasing fine organic sediment deposition would be associated with declines in macroinvertebrate density and taxonomic richness in both the experimental systems and the field survey of peatland rivers, driven primarily by losses of sensitive taxa such as Ephemeroptera and Plecoptera (Brown et al., 2015; Larsen & Ormerod, 2010b); (H_2_) these changes would be attributable to species sorting (cf. environmental filtering) processes acting on whole suites of traits, reflecting the selection of different life strategies under increasing levels of fine organic sediment deposition (Verberk, Noordwijk, & Hildrew, 2013), rather than through a trait–environment relationship characterized by simple or additive associations (Wilkes, Mckenzie, Murphy, & Chadd, 2017); (H_3_) similar responses would be evident between controlled experiments and headwater rivers as a consequence of the physical effects of sedimentation on aquatic biota.

## 2 MATERIALS AND METHODS

The study was undertaken in March and April 2010 in the Pennine hills of northern England (Supporting Information Figure S1). The Pennines cover >31,000 km^2^ and stretch from the Peak District in the south through the Yorkshire Dales to the North Pennines Area of Outstanding Natural Beauty. The high plateaus and valleys of the Pennines support extensive areas of blanket bog and valley mire. Our study in this region comprised two linked pieces of work: (a) a riverside mesocosm experiment and (b) a survey of headwaters representative of those draining blanket peatlands across the Pennines.

### 2.1 Mesocosm experiment

The mesocosm experiment was undertaken in a facility comprising 24 channels located alongside Moss Burn, a second‐order tributary of Trout Beck, within the Moor House National Nature Reserve, northern England (Table 1; Supporting Information Figure S1). Moss Burn is a stony‐bed river flowing across open blanket peat moorland. Owing to the upland peat‐dominated soil cover, Moss Burn has a flashy flow regime (i.e. short lag times between peak rainfall and peak runoff) similar to all of the field survey sites described below, characterized by high flows reaching >2 m^3^/s and then prolonged periods of base flow (<0.2 m^3^/s).

**Table 1:** Summary information for sites sampled in the peatland rivers survey. [See Supporting Information Figure S1 for map]

River	Lat/Long	Catchment area (km^2^)	Catchment altitude (m AOD)	Geology
Bull Clough	53°28′24.8″N; 1°42′46.2″W	0.7	455–541	Carboniferous and Jurassic sandstone
Crowden Little Brook	53°30′51.7″N; 1°53′29.7″W	3.1	355–582	Carboniferous gritstone and sandstone
Great Eggleshope Beck	54°40′59.6″N; 2°04′11.9″W	1.6	480–653	Carboniferous mudstone, sandstone and limestone
Green Burn	54°40′40.0″N; 2°21′43.9″W	0.7	548–734	Carboniferous sandstone, limestone and shale
Lodgegill Sike	54°40′35.5″N; 2°04′04.1″W	1.2	515–608	Carboniferous mudstone, sandstone and limestone
Moss Burn	54°41′19.7″N; 2°23′01.7″W	1.4	560–768	Carboniferous sandstone, limestone and shale
Oakner Clough	53°36′11.1″N; 1°58′03.4″W	1.2	240–451	Carboniferous gritstone and sandstone
Rising Clough	53°23′38.4″N; 1°40′25.0″W	1.8	344–487	Carboniferous gritstone and sandstone
Trout Beck	54°40′59.6″N; 2°24′46.0″W	2.8	595–794	Carboniferous sandstone, limestone and shale
Woo Gill	54°12′06.1″N; 1°53′26.3″W	1.0	430–546	Carboniferous and Jurassic mudstone

River water was diverted from Moss Burn through 9 × 68 mm diameter pipes which sampled water from different depths prior to the river cascading over small bedrock falls. Flow along the pipes was controlled using a series of valves, and water was transferred approximately 20 m downstream under gravity to three header tanks to buffer inflowing coarse sediment and flow pulses. Each header tank subsequently fed a block of eight mesocosm channels. Crawling invertebrates could emigrate from tanks via outflow pipes using mesh ladders that were connected to the tank floor. Each of the 24 individual mesocosm channels was 1 m (L) × 0.1 m (W) × 0.1 m (D). Mesocosm channels were constructed from guttering mounted on wooden frames, with inflows and outflows constructed from 32‐mm pipe. Valves were used to equalize the inflow of water from header tanks to each mesocosm. All channels were filled with sediment from Moss Burn to a depth of ~5 cm, with the same proportions of silt, gravel, pebbles and cobbles added to each mesocosm. Mean water depth in each channel was ~5 cm, and discharge was 0.3 L/s. An open outflow pipe at the end of the channels allowed the natural drainage of water and emigrating biota back to Moss Burn to prevent cross‐colonization.

The mesocosm experiment ran for 4 weeks (28 days). Sediment treatments were established on day 1, mimicking a pulse of sediment deposition on riverbed habitat patches that occur in eroding peatlands due to disturbances such as riverbank failures (Crowe & Warburton, 2007), and larger hillslope slumps or slides (Dykes & Selkirk‐Bell, 2010; Kokelj et al., 2013). Macroinvertebrates then colonized the mesocosms via drift, swimming, crawling and aerial oviposition over the 4‐week period. This length of time was required in previous experiments (conducted in April 2009) for invertebrate communities to develop no differences across individual mesocosm channels (Brown, unpublished data). Disaggregated peat sieved to <1 mm was added to mesocosm channels to create fine particulate organic matter (FPOM) treatments. The ratio of organic and inorganic sediments within the channel varied depending on the treatment to represent a gradient of organic benthic sediment densities: (a) control, having no organic sediment added as substrate in the channel, (b) 25% of bed area as organic substrate (~2.5 [±0.07 *SE*] g/m^2^ ash‐free dry mass; ~225 g/m^2^ peat addition), (c) a 50% organic treatment (~5.0 [±0.14] g/m^2^; ~450 g/m^2^ peat addition) and (d) 75% treatment (~7.5 [±0.20] g/m^2^; ~675 g/m^2^ peat addition). The volume of peat added relative to AFDM estimates is a function of the high water and organic matter content of peat. The upper density was within the range of observations made during previous surveys of UK peatland rivers (Brown et al., 2013). Each treatment was replicated six times.

Water temperature, electrical conductivity (EC), dissolved oxygen (DO) and pH were measured weekly in each of the channels and the source river using a Hach HQ40d portable multi‐parameter meter. Water was collected from each channel and the river in the final week of the experiment to examine effects of sedimentation on suspended sediment concentrations (SSC), dissolved organic carbon (DOC) and total oxidized nitrogen (TON) which have been shown to increase in response to peat inputs to rivers in other studies (Aspray et al., 2017; Daniels, Evans, Agnew, & Allott, 2012). Water samples (500 ml) were filtered, dried and weighed to determine SSC, while water samples for DOC and TON were passed through 0.45‐μm Whatman cellulose filters prior to analysis with a Thermalox 8000 total carbon analyser and a Skalar SAN++ continuous flow analyser, respectively. Each channel was sampled in its entirety for macroinvertebrates at the completion of the experiment by elutriating sediments through a Surber net (250‐μm mesh). All macroinvertebrate samples were preserved immediately in 70% ethanol and later sorted and identified in the laboratory.

### 2.2 Peatland river survey

Concurrently with the mesocosm experiment, ten headwater rivers were sampled in upland areas >290 m altitude and with catchment sizes all <3.1 km^2^ during March 2010 (Table 1). River size was similar throughout the sites and those chosen for study comprised second‐ or third‐order rivers, determined from 1:25,000 Ordnance Survey (OS) maps. Vegetation in all catchments was predominantly *Calluna vulgaris*, *Vaccinium myrtillus*, *Sphagnum* spp. and *Eriophorum* spp. with *Juncus* spp. also present in the riparian zone. The study rivers drained catchments with light sheep grazing (<1 ewe per ha) management, areas of bare exposed peat and/or rotational vegetation burning management, typical of upland peatland systems in the United Kingdom. In the burned catchments, recent burn patches (<2 years since burning) were predominantly exposed peat with only a small cover of mosses and *Calluna* shoots. Older burn patches (>5 years since burning) were dominated by *Calluna* at various stages of growth. All catchments exhibited localized river bank erosion, typically with exposed peat on river banks, providing additional sources of fine particulates to rivers. A single study reach of ~25 m, possessing riffle, glide and run habitats and with minimal direct shading from vegetation, was selected randomly in each study river for detailed macroinvertebrate biodiversity studies.

Water temperature, EC and pH were measured on site, and water samples were collected for SSC, DOC and TON analysis, following the same methods as in the mesocosm experiment described above. Five benthic macroinvertebrates samples were collected at each river using a modified Surber sampler (0.05 m^2^ area; 250‐μm mesh). Samples were preserved immediately in 70% ethanol and later sorted and identified in the laboratory. From each Surber sample, benthic particulate organic matter (POM) was retained. The fine (<1 mm; FPOM) fraction was oven‐dried and ashed to determine ash‐free dry mass per m^2^ (i.e. benthic peat sedimentation density).

### 2.3 Data analysis

Macroinvertebrates were identified to species level where possible, and genus in most other cases, using standard keys detailed in Pawley, Dobson, and Fletcher (2011). Chironomidae larvae were identified to family and Oligochaeta to class. Analyses were undertaken at four levels of organization: (A) *Benthic macroinvertebrate community*, based upon the following metrics: (i) total macroinvertebrate density (per m^2^), (ii) richness (n taxa), (iii) beta diversity (Sørensen index) amongst replicate samples (i.e. mesocosms for each experimental treatment, Surber samples for each headwater river), with partitioning analysis to consider elements of turnover (species replacements between sites) and nestedness (species loss from site to site; Baselga & Orme, 2012); (B) *Order level*, with densities calculated for the Chironomidae, Ephemeroptera, Plecoptera and Coleoptera, which are typically the most common macroinvertebrate orders in peatland river systems; (C) *Species level*: densities were calculated for *Leuctra inermis*, typically the most common Plecoptera species found in peatland rivers but which is known to be sensitive to sedimentation effects in rivers (Turley et al., 2016); and (D) *Traits and functional diversity*: we used the same traits from previous assessments of fine sediment effects on river invertebrates (Wilkes et al., 2017) to enable a clearer understanding of their links with organic sediments. Traits were assigned to invertebrate genera using the fuzzy codes (Chevenet, Dolédec, & Chessel, 1994) from the database developed by Tachet, Richoux, Bournaud, and Usseglio‐Polatera (2010) (see Supporting Information Table S2 for traits used, their modalities and codes). Taxon densities (untransformed) were used to create a density‐weighted trait matrix [samples × traits]. From this, we assessed sedimentation effects on individual traits, community‐level trait profiles and functional diversity (FD). FD was assessed in terms of functional richness (FRic; proportion of functional space filled by a community) and functional dispersion (FDis; the density‐weighted deviation of species trait values from the centre of the functional space). These two FD indices were chosen to represent the effects of presence–absence (FRic) and abundance (FDis) structure on the distribution of communities in functional space (Mouillot, Graham, Villéger, Mason, & Bellwood, 2013). FRic and FDis have previously been shown to respond in a strong and consistent way to habitat gradients in headwater rivers globally (Brown et al., 2018).

All statistical analyses were undertaken using r 3.4.3. Beta diversity partitioning was undertaken using the betapart package (Baselga & Orme, 2012). FD indices were generated using the dbFD function in the fd package (Laliberté & Legendre, 2010). For the mesocosm experiment, linear mixed‐effects (lme) models were used to analyse invertebrate biodiversity metric responses to FPOM (fixed effect), with combinations of block, replicate and block/replicate incorporated as random effects. Akaike Information Criterion scores were calculated to determine the most parsimonious model. Mixed‐effect models typically performed better than models incorporating only fixed effects, but because no combination of effects was consistently the best performing, we adopted the model: Response ~FPOM + (1|block/replicate) for all subsequent analyses. Models were fitted using the nlme package (Pinheiro, Bates, Debroy, & Sarkar, 2006), with marginal *R* ^2^ values calculated following Nakagawa, Schielzeth, and O'Hara, (2013) as implemented in the MuMIn package (Barton, 2013). For the field survey data, we used linear models (lm) to test for fixed effects of FPOM on invertebrate biodiversity metric responses. Linear models were used after testing for a range of potential distributions with maximum‐likelihood estimates on residuals of linear models using the mass package (Venables & Ripley, 2002). Trait–environment associations were assessed using the ade 4 package (Dray & Dufour, 2007). The fourth corner method (Legendre, Galzin, & Harmelin‐Vivien, 1997) was used to test for significant one‐to‐one correlations between experimental treatment and individual traits, whereas the RLQ method (Dray et al., 2014) was used to examine the significance of the overall link between all traits and the environment in (a) the mesocosms and the river sites combined, and (b) the mesocosms alone, given the latter afforded experimental isolation of sediment deposition effects.

## 3 RESULTS

### 3.1 Mesocosm experiment

During the experiment, Moss Burn averaged 0.13 (±0.001 *SE*) m depth, with a mean discharge of 0.08 (±0.003) m^3^/s measured at a rated cross section located adjacent to the mesocosm inflow pipes. Discharge in the mesocosms remained stable throughout the treatments and the course of the experiment (mean 0.0003 ± 0.00009 m^3^/s). DO, pH, water temperature and EC were very similar amongst mesocosms, and to Moss Burn, throughout the experiment (Table 2; Supporting Information Table S1). SSC and DOC sampled at the end of the experiment showed minimal variation and no significant difference between treatments, but TON increased significantly with benthic sediment cover (Table 2). Forty‐seven macroinvertebrate taxa colonized the mesocosm array.

**Table 2:** Descriptive statistics and model results for physicochemical variables measured at the end of the mesocosm experiment and in the peatland river survey

	Temperature (°C)	pH	EC (µS/cm)	SSC (mg/L)	DOC (mg/L)	TON (mg/L)
Mesocosms						
Control						
Mean	7.0	7.4	63.9	1.24	7.0	0.07
Median	7.0	7.4	63.9	1.43	6.67	0.08
Max	7.0	7.6	64.0	2.00	10.42	0.10
Min	7.0	7.1	63.7	0.20	4.71	0.04
2.5 g/m						
Mean	7.1	7.4	63.8	1.42	10.17	0.07
Median	7.1	7.4	63.9	1.16	5.73	0.08
Max	7.1	7.5	63.9	3.77	32.0	0.09
Min	7.0	7.2	63.7	0.17	5.11	0.06
5.0 g/m						
Mean	7.0	7.4	63.8	1.28	7.83	0.09
Median	7.0	7.4	63.8	1.11	6.41	0.10
Max	7.1	7.6	64.1	3.06	12.24	0.11
Min	7.0	7.2	63.3	0.19	5.13	0.08
7.5 g/m						
Mean	7.1	7.4	63.8	0.11	6.42	0.91
Median	7.1	7.4	63.9	0.10	6.39	0.93
Max	7.1	7.6	63.9	0.20	10.11	1.80
Min	7.0	7.3	63.6	0.08	2.13	0.38
Lme summary	*t* = 1.44	*t* = 1.03	*t* = 1.34	*t* = −0.64	*t* = −0.45	*t* = 2.95
	*R* ^2^_m_ = 0.08	*R* ^2^_m_ = 0.004	*R* ^2^_m_ = 0.058	*R* ^2^_m_ = 0.018	*R* ^2^_m_ = 0.006	*R* ^2^_m_ = 0.27
	*p* = 0.17	*p* = 0.33	*p* = 0.20	*p* = 0.53	*p* = 0.65	***p* = 0.009**
River survey						
Mean	12.5	5.5	59.1	6.65	11.75	1.02
Median	11.0	5.6	55.5	5.65	11.29	0.98
Max	18.0	7.6	105.0	17.00	23.49	1.74
Min	8.7	3.0	33.0	1.70	1.24	0.16
Lm summary	*t* = −1.01	*t* = −0.31	*t* = 1.04	*t* = 1.04	*t* = 1.02	*t* = 2.06
	*R* ^2^ = 0.11	*R* ^2^ = 0.01	*R* ^2^ = 0.12	*R* ^2^ = 0.12	*R* ^2^ = 0.12	*R* ^2^ = 0.35
	*p* = 0.34	*p* = 0.77	*p* = 0.33	*p* = 0.33	*p* = 0.34	*p* = 0.07

Note

Significant *p* values highlighted in bold. [See Supporting Information Table S3 for summary statistics for all models; lme results for mesocosms incorporate random effects of replicate in block]

**Figure 1:**
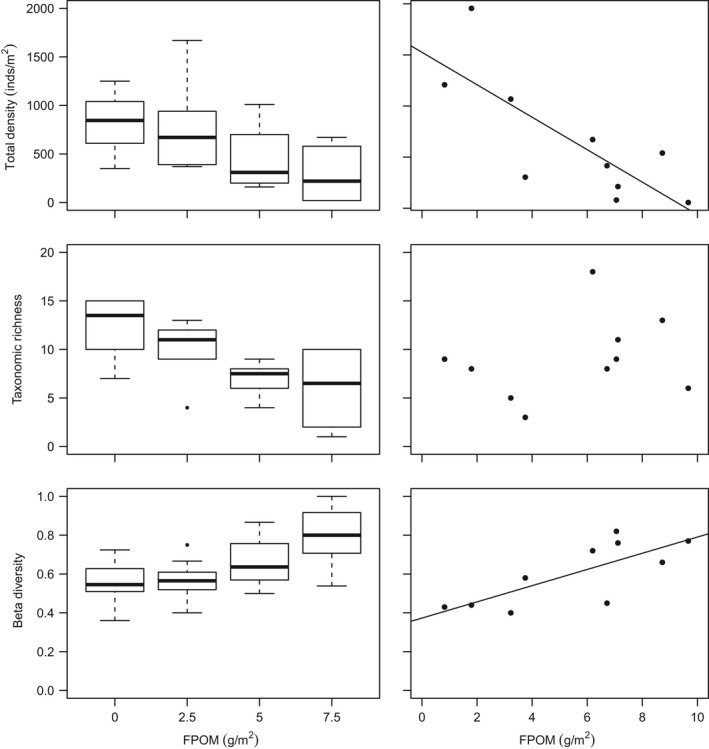
Community‐level responses to organic sedimentation for (left) the mesocosm experiment and (right) peatland headwater surveys

**Figure 2:**
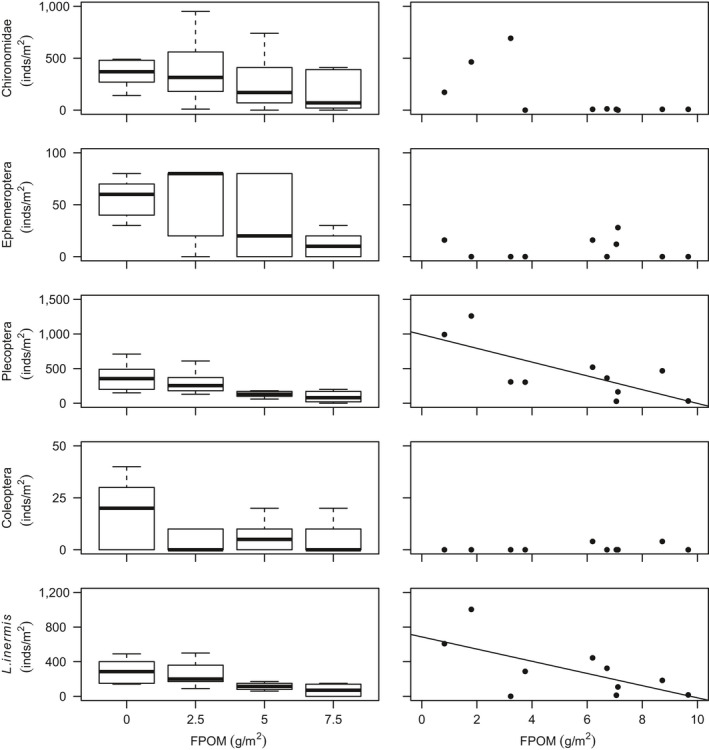
Taxonomic responses to organic sedimentation for (left) the mesocosm experiment and (right) peatland headwater surveys

The invertebrate community was dominated by Chironomidae, Plecoptera and Ephemeroptera taxa (Table 3; Supporting Information Figure S2) predominately due to high densities of *Leuctra* spp. (mainly *Leuctra inermis*), and *Baetis* spp. Other common taxa included stoneflies such as *Amphinemura* spp., *Nemoura *spp., *Ameletus inopinatus*, *Chloroperla torrentium* and *Isoperla grammatica*. No significant differences between control vs. 2.5 g/m treatment were found for any biodiversity variables (Supporting Information Table S4). However, the density of macroinvertebrate assemblages in the mesocosm channels decreased by 65% on average with benthic sedimentation (control vs. 7.5 g/m^2^ treatment), and taxonomic richness decreased by 50%, whereas beta diversity increased (Table 3; Figure 1). Sedimentation showed no relationship with turnover beta diversity (*R* ^2^_m_ = 0.02, *p* = 0.24), but there was a weak association with nestedness (*R* ^2^_m_ = 0.07, *p* = 0.037; Supporting Information Table S3). Significantly lower overall density and taxonomic richness observed in higher bed sedimentation treatments were driven mainly by losses of Plecoptera, and to a lesser extent Ephemeroptera (Table 3; Figure 2). Within the Plecoptera, *L. inermis* density declined markedly as sedimentation increased.

**Table 3:** Descriptive statistics and model results for macroinvertebrate community metrics and taxonomic‐level responses measured at the end of the mesocosm experiment and in the peatland river survey

	Density (inds. per m^2^)	Taxonomic Richness	Beta diversity	Chironomidae (inds. per m^2^)	Ephemeroptera (inds. per m^2^)	Plecoptera (inds. per m^2^)	Coleoptera (inds. per m^2^)	*L. inermis *(inds. per m^2^)	Shredder (inds. per m^2^)	FRic	FDis
Mesocosms											
Control											
Mean	823	12	0.55	353	57	377	18	290	129	0.38	6.35
Median	845	14	0.55	370	60	355	20	290	120	0.38	6.33
Max	1,250	15	0.72	490	80	710	40	490	237	0.76	6.57
Min	350	7	0.36	140	30	150	0	140	53	0.07	6.26
2.5 g/m^2^											
Mean	785	10	0.57	388	57	300	3	250	100	0.31	6.09
Median	670	11	0.57	315	80	255	0	200	83	0.28	6.30
Max	1,670	13	0.75	950	80	610	10	500	203	0.66	7.25
Min	370	4	0.40	10	0	130	0	90	43	0.04	4.77
5.0 g/m^2^											
Mean	448	7	0.66	260	33	128	7	120	46	0.27	5.52
Median	310	8	0.64	170	20	130	5	120	45	0.17	5.66
Max	1,010	9	0.87	740	80	180	20	170	60	0.64	6.37
Min	160	4	0.50	0	0	60	0	60	23	0.01	4.42
7.5 g/m^2^											
Mean	288	6	0.80	160	12	92	5	70	32	0.37	4.66
Median	220	7	0.80	70	10	80	0	70	27	0.36	5.76
Max	670	10	1.00	410	30	200	20	150	67	0.62	8.16
Min	20	1	0.54	0	0	0	0	0	0	0.14	0
lme summary	*t* = −3.32	*t* = −4.63	*t* = 5.74	*t* = −1.82	*t* = −3.10	*t* = −4.54	*t* = −1.87	*t* = −4.51	*t* = −4.77	*t* = −0.53	*t* = −2.30
	*R* ^2^_m_ = 0.28	*R* ^2^_m_ = 0.39	*R* ^2^_m_ = 0.36	*R* ^2^_m_ = 0.10	*R* ^2^_m_ = 0.30	*R* ^2^_m_ = 0.41	*R* ^2^_m_ = 0.13	*R* ^2^_m_ = 0.42	*R* ^2^_m_ = 0.42	*R* ^2^_m_ = 0.01	*R* ^2^_m_ = 0.14
	***p* = 0.004**	***p* = 0.0002**	***p* < 0.00001**	*p* = 0.09	***p* = 0.007**	***p* = 0.0003**	*p* = 0.08	***p* = 0.0003**	***p* = 0.0002**	*p* = 0.60	***p* = 0.034**
River survey											
Mean	651	9	0.48	137	7	444	1	299	445	0.24	5.00
Median	478	9	0.50	8	0	336	0	236	342	0.15	4.44
Max	1,956	18	0.79	692	28	1,260	4	1,004	1,260	0.76	9.83
Min	56	3	0.20	0	0	28	0	0	24	0.002	0.56
lm summary	*t* = −3.54	*t* = 0.83	*t* = 4.08	*t* = −2.23	*t* = −0.003	*t* = −3.07	*t* = 1.05	*t* = −2.43	*t* = −3.09	*t* = 2.04	*t* = 0.99
	*R* ^2^ = 0.61	*R* ^2^ = 0.08	*R* ^2^ = 0.68	*R* ^2^ = 0.38	*R* ^2^ = 0.0001	*R* ^2^ = 0.54	*R* ^2^ = 0.12	*R* ^2^ = 0.43	*R* ^2^ = 0.54	*R* ^2^ = 0.34	*R* ^2^ = 0.11
	***p* = 0.008**	*p* = 0.43	***p* = 0.004**	*p* = 0.06	*p* = 0.99	***p* = 0.015**	*p* = 0.32	***p* = 0.041**	***p* = 0.015**	*p* = 0.08	*p* = 0.35

Note

Significant *p* values highlighted in bold. [See Supporting Information Table S3 for summary statistics for all model; lme results for mesocosms incorporate random effects of replicate in block. See Supporting Information Table S4 for mesocosm pairwise comparisons].

**Figure 3:**
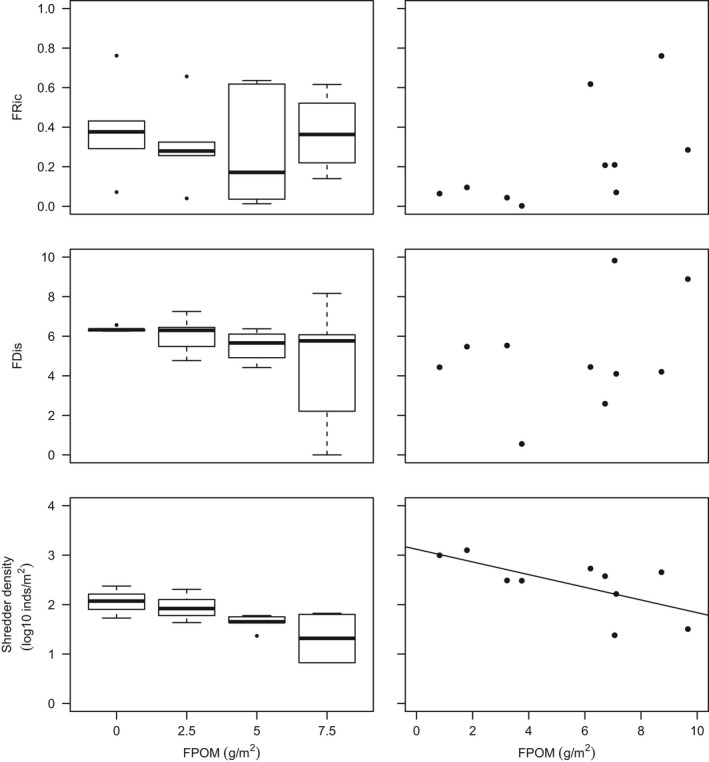
Functional richness (FRic), functional dispersion (FDis) and shredder densities for (left) the mesocosm experiment and (right) peatland headwater surveys

**Figure 4:**
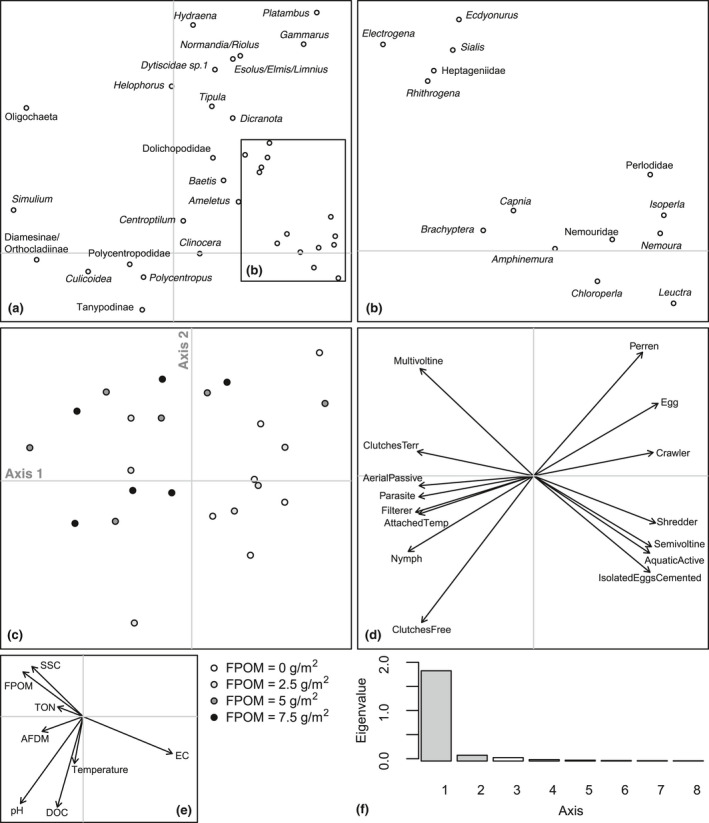
Results of the first two axes of the RLQ analysis on mesocosm experiment communities only: (a) species scores; (b) species scores not labelled in the inset shown in (a); (c) replicate mesocosm scores with shading referring to treatment; (c) coefficients for key environmental variables; (d) coefficients for those traits with the strongest link to environment (see Supporting Information Table S2 for trait codes and Supporting Information Table S5 for *p* values); (e) coefficients for key environmental variables; and (f) eigenvalues for axes 1–8. Abbreviations: ash‐free dry mass (AFDM); dissolved organic carbon (DOC); electrical conductivity (EC); fine particulate organic matter (FPOM); total oxidized nitrogen (TON); and suspended sediment concentration (SSC)

**Figure 5:**
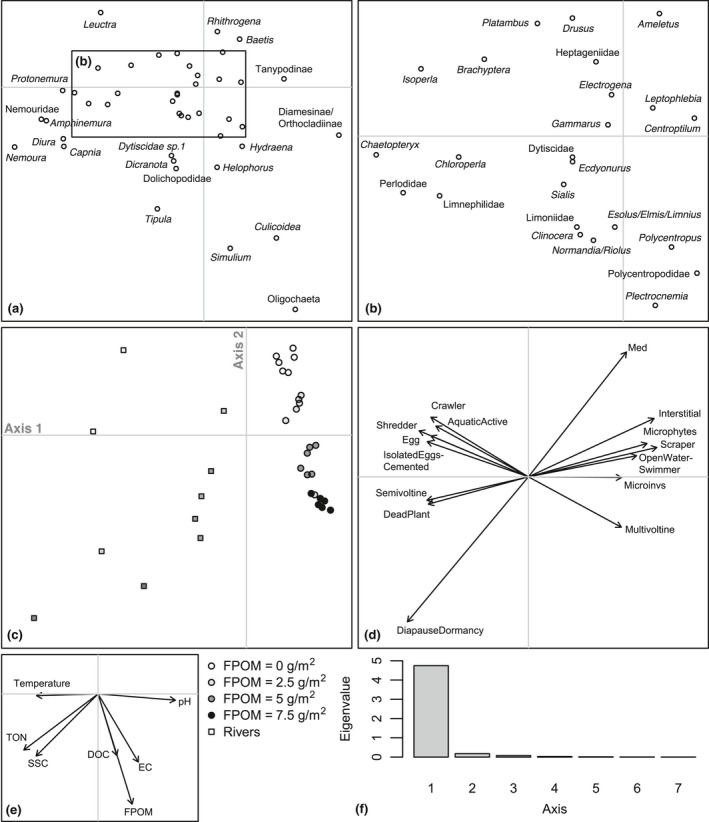
Results of the first two axes of the RLQ analysis on mesocosm experiment and peatland headwater surveys communities combined: (a) species scores; (b) species scores not labelled in the inset shown in (a); (c) site scores with darker symbols denoting increasing sedimentation in mesocosms (circles) and headwater sites (squares); (d) coefficients for those traits with the strongest link to environment (see Supporting Information Table S2 for trait codes and Supporting Information Table S5 for *p* values); (e) coefficients for key environmental variables; and (f) eigenvalues for axes 1–7. Abbreviations as per Figure 4

There was no clear tendency for FRic to decrease with benthic sedimentation. However, as sedimentation increased, FDis became more variable between replicates within treatments and mean FDis decreased (Table 3; Figure 3). The fourth corner analysis revealed that there were no significant one‐to‐one trait–environment relationships amongst the experimental communities (Supporting Information Table S5) although a significant negative response of taxa with a strong affinity to shredding feeding habit was evident (Table 3; Figure 3). The overall link between multiple traits and sedimentation in mesocosms was significant (RLQ: *p* = 0.04). A single axis dominated the variability between communities in multivariate trait space amongst treatments (Figure 4) and when mesocosms and headwater surveys were combined in the RLQ analysis (Figure 5). The combined RLQ analysis emphasized the effectiveness of the experimental control, with mesocosms clustered along axis 1 relative to headwaters, and with treatments arrayed along axis 2 in relation to FPOM density. Along the gradient of sedimentation, life strategies based on longer life cycles (univoltine), active aquatic dispersal of larvae (including crawling), fixed aquatic eggs and shredding feeding habits were replaced by resilient and resistant strategies based on multivoltinism, temporary attachment and avoidance of impacts on eggs through terrestrial oviposition, as well as fine detritus diets and filter‐feeding habits (Figure 4).

### 3.2 Peatland river survey

No significant relationships were found between benthic FPOM density and the physicochemical variables monitored during the field survey, although TON showed a positive association which was only marginally insignificant (Table 2). Thirty‐three macroinvertebrate taxa were collected during the study, dominated by Chironomidae, Plecoptera and Ephemeroptera (Table 3; Supporting Information Figure S2). Common taxa included *L. inermis*, *Nemoura* and *Amphinemura standfussi*. Overall macroinvertebrate density decreased by 95% across the FPOM density gradient, beta diversity increased, but there was no relationship with taxonomic richness (Table 3; Figure 1). In contrast to the mesocosm experiment, FPOM density was associated with turnover beta diversity (*R* ^2^ = 0.41, *p* = 0.047) but not with nestedness (*R* ^2^ = 0.02, *p* = 0.73).

Significant decreases in Plecoptera density, including *L. inermis*, were observed with increasing benthic FPOM density (Table 3; Figure 2). No significant relationships were found between FPOM density and FRic or FDis (Figure 3), although notably FDis varied more for all sites >5 g/m^2^ (range = 7.22) compared with sites <5 g/m^2^ (range = 4.99). In the combined RLQ analysis, headwater communities contrasted with those from the mesocosm experiment along a gradient closely corresponding to voltinism, with disturbance‐tolerant, multivoltine taxa preferentially colonizing mesocosms (Figure 5). However, the overall link between traits and the environment was not significant when experimental and river communities were combined (RLQ: *p* = 0.41).

## 4 DISCUSSION

Changing land use and land management, and the effects of acidification and climate change, have contributed to elevated sedimentation in river networks around the world (Piggott et al., 2015; Wood & Armitage, 1997). Organic‐rich sediment loss from peatlands is forecast to increase by typically around 14% by 2100 under climate change due to increasing temperature and enhanced occurrence of summer desiccation (Li, Irvine, et al., 2017b). However, there will be a high variability in sediment loads from peatlands. Some headwater peatland regions may move out their current bioclimatic envelopes and be more at risk to enhanced erosion (Clark et al., 2010; Gallego‐Sala & Prentice, 2012), and some sites have been forecast to have more than double their current annual sediment loads by 2100 (Li et al., 2016; Li, Irvine, et al., 2017b). Organic sedimentation of headwater rivers will therefore increase in the future, and for the first time, our study shows how these sediments can be expected to influence aquatic ecosystems that receive runoff from blanket peatlands. In particular, we identified previously unknown structural and taxonomic responses that can be developed further as indicators of peatland river sedimentation stress. We also illustrate that increasing organic sedimentation can drive more stochastic assembly processes, but at a higher threshold of deposition before effects are seen compared with evidence from inorganic sediment experiments. Importantly, our study provides clear experimental evidence that organic sediment is a significant stressor for aquatic biodiversity to corroborate correlative field survey results obtained from rivers influenced by real‐world land management gradients. These major findings are discussed in turn below.

### 4.1 Community and taxonomic responses

Complementary approaches of mesocosm experiments and river surveys in temperate‐zone northern peatlands provide new evidence that benthic sedimentation from peat erosion causes significant alterations to invertebrate biodiversity in the headwaters of river systems. Consistent reductions in overall density, reduced taxonomic richness in the experimental mesocosms, and similar increases in beta diversity for both study systems supported the first part of H_1_, that sedimentation from eroding peatlands serves to alter headwater invertebrate community biodiversity. These results are supported by field surveys that have implicated organic sediment deposits as a major driver of aquatic biodiversity change following peatland management by artificial drainage and catchment restoration via drain blocking (Ramchunder, Brown, & Holden, 2012), vegetation burning (Brown et al., 2013; Ramchunder, Brown, & Holden, 2013) and forestry (Vuori & Joensuu, 1996). Similarly, our finding that overall densities of invertebrates declined with sedimentation is consistent with field studies that have demonstrated links between the slumping of Arctic permafrost soils linked to climate warming, deposition of fine sediments in river systems and aquatic biota responses (Chin et al., 2016). Together, these different studies point towards elevated fine particulate organic sediments serving as a major stressor for aquatic invertebrate in a range of headwater systems draining peat‐dominated landscapes.

Declines in invertebrate density and taxonomic richness were accompanied by significant changes in species composition, driven predominantly by losses of Plecoptera (and Ephemeroptera in mesocosms), further supporting H_1_ and as observed in studies of inorganic sedimentation (Angradi, 1999; Larsen et al., 2009; Wood, Toone, Greenwood, & Armitage, 2005). Similar to our findings, many species from these Orders have frequently been reported to have a low tolerance of fine sediments in both experimental studies (Larsen & Ormerod, 2010b; Matthaei, Weller, Kelly, & Townsend, 2006) and field surveys (Larsen et al., 2009; Richards & Bacon, 1994). *L. inermis* was particularly dominant in the mesocosm control channels but showed one of the largest declines in density as sedimentation increased, with these responses mirrored along the deposited sediment gradient in headwater rivers. Declines in *L. inermis* density can be linked to its known high sensitivity to sedimentation (Extence et al., 2013; Turley et al., 2016). Previous short‐duration (1 day) sediment pulse experiments in Moss Burn demonstrated rapid behavioural drift of *Leuctra* (Aspray et al., 2017) to avoid sediment deposition in the benthos, most likely due to sediment smothering causing significant reductions in the delivery of oxygenated water into interstitial habitats.

Despite most previous sedimentation experiments predominantly reporting invertebrate responses to inorganic material as opposed to organic sediments, it is likely that the changes observed in river invertebrates in our study were provoked by similar drivers such as clogging of interstitial spaces, associated reductions in pore water DO concentrations, and a loss of habitat and refuge availability (Aspray et al., 2017; Jones et al., 2012; Larsen et al., 2009). Compared to our study of organic sedimentation, where significant effects were found only in mesocosm treatments above 5 g/m^2^ bed cover (corresponding to ~50% cover), some experiments utilizing inorganic sediments have found significant effects at lower levels (33% cover; Angradi, 1999; Larsen & Ormerod, 2010b). This could be due to a subsidy–stress effect, whereby river ecosystems have increased tolerance to organic sediments compared to inorganic sediments at low/intermediate densities due to beneficial effects such as nutrient retention and a food subsidy for invertebrates (Aldridge et al., 2009; Peeters et al., 2006). When organic sedimentation reaches a specific level or tipping‐point, it may then begin to act more as a stressor.

In addition to the direct physical effects of sedimentation on invertebrates, our study revealed organic sediment deposition as a nutrient source, with TON concentrations increasing significantly in treatments with higher benthic organic sediment and a similar (although marginally insignificant) response observed across headwater rivers. Nitrogen dynamics have long been considered to be influenced heavily by sediment influx in peatland catchments, with Crisp (1966) suggesting 80% of nitrogen output in an upland headwater river was a consequence of peat erosion, and Daniels et al. (2012) showed that NH_4_ released from eroded peat was nitrified rapidly. These nutrient subsidies might drive alterations to river metabolic processes in otherwise low‐productivity peatland river systems (Aspray et al., 2017). This points towards a need for more peatland river studies to understand the nature of interacting multiple stressors, in a manner similar to experimental manipulations that have uncovered biodiversity responses to sediment interactions with nutrients, flow and temperature alterations in lowland agricultural settings (Matthaei, Piggott, & Townsend, 2010; Piggott et al., 2015).

### 4.2 Traits and functional diversity

Increasing organic sedimentation was not accompanied by a change in functional richness despite driving clear reductions in taxonomic richness, suggesting functional redundancy amongst peatland river invertebrate communities. While functional richness highlighted similar trait “volumes” amongst mesocosm treatments and along the headwater river sedimentation gradient, a shift in the volume centroid was corroborated by the RLQ results. Although the overall trait–environment link was marginally insignificant for the combined RLQ analysis, the mesocosm‐only analysis showed a significant relationship. This highlights the benefit of mesocosms for experimentally controlling confounding variables to enable a direct analysis of trait‐sedimentation responses. Mesocosm results suggested invertebrate community changes can be partially attributed to species‐sorting processes acting on whole suites of traits that each organism possesses, reflecting the selection of disturbance‐tolerant “life strategies” under increasing levels of fine organic sediment deposition (Verberk et al., 2013) as expected for H_2_, rather than through a trait–environment relationship characterized by simple or additive associations (Wilkes et al., 2017). While the overall suite of traits responding to organic sedimentation was not directly comparable to those observed in previous studies (in part due to different profiles of traits used for analyses within different studies), some of our key findings are supported by previous studies from different geographical locations. For example, sedimentation may select for taxa with shorter life cycles that are resilient to disturbances, as well as taxa with fine detritus deposit/suspension feeding habits which are dependent on fine sediment as a food resource (Buendia, Gibbins, Vericat, Batalla, & Douglas, 2013; Larsen & Ormerod, 2010a; Wagenhoff, Townsend, & Matthaei, 2012). Additionally, taxa considered to be shredders may be selected against in a process thought to be related to burial of leaf litter and reductions in its nutritional quality due to the inhibition of fungal growth (Descloux et al., 2014; Larsen & Ormerod, 2010a; Vuori & Joensuu, 1996; Wilkes et al., 2017). Taxa with a propensity for crawling as a method of locomotion may also be negatively impacted due to their relatively slow movement rates leaving them susceptible to burial (Buendia et al., 2013).

Functional dispersion patterns suggested less abundance‐weighted separation of peatland river invertebrates with increasing sediment deposition in both the mesocosms and headwaters survey, consistent with only certain trait combinations conferring tolerance. The higher variability in functional dispersion concurrent with increasing taxonomic beta diversity as sediment deposition increased in both systems leads us to hypothesize that stochastic components of community assembly increase with higher organic sediment content. These results correspond with studies of other freshwater ecosystem stressors (Chase, 2010) where priority effects were stronger in more productive environments. Similar interpretations of invertebrate community responses to elevated sand deposition in the regulated River Usk, Wales, were proposed by Larsen and Ormerod (2014) but based on species co‐occurrence rather than functional diversity methods. Nevertheless, the similarity of these independent findings should serve to encourage future aquatic sedimentation studies to determine whether consistent assembly processes (Brown et al., 2018) are evident across sedimentation‐impacted rivers in different locations.

### 4.3 Using mesocosms to understand headwater river biodiversity response to stressors

The experimental mesocosm channels and their source river, Moss Burn, showed consistently similar physicochemical characteristics that were also congruent between replicates, meaning conditions between mesocosm blocks were both realistic and replicable. While the mesocosms were colonized by more taxa than we found in the headwaters survey, many of these extra taxa were single individual occurrences likely reflecting the larger number of replicates collected in the mesocosm array compared with individual headwater rivers, plus potentially more flow disturbances in headwater rivers compared to constant flows through the mesocosms. Nevertheless, mesocosms and headwater river samples were both dominated heavily by Chironomidae and Leuctridae. This direct source vs. mesocosm comparison supports the general contention that riverside mesocosms can provide realistic environments for experimental manipulation (Ledger, Harris, Armitage, & Milner, 2009). However, upscaling experimental results to inform wider headwater river network biodiversity patterns and processes requires comparisons across multiple rivers (Larsen et al., 2009). RLQ results showed that, for many rivers, invertebrate trait–environment links were much broader than those in the mesocosms. A particularly lower representation of longer‐lived taxa in mesocosms was perhaps a consequence of colonization for these groups being restricted during the short‐duration experiment, or because the controlled experimental conditions (e.g. flow rates, depth, sediments) mimicked only a small fraction of habitat patches found in river networks. The mesocosm communities were also positioned at the positive region of axis one, with cooler water temperatures likely to have further contributed to fewer long‐lived taxa. Some rivers also had much higher SSC and TON concentrations, perhaps due to larger expanses of eroding peat in their catchments than is the case at Moss Burn. Despite these differences amongst rivers, there was a clear arrangement of rivers along axis 2 similar to the mesocosms, and with a clear association with FPOM concentration.

The results highlighted comparable responses to sedimentation (i.e. significant and non‐significant) for eight of 11 measures of invertebrate biodiversity in mesocosms and headwater rivers, providing support for H_3_. Despite the potential for confounding effects of other environmental variables influencing invertebrates in headwater rivers (Wagenhoff et al., 2012), the similarity of headwater invertebrate community responses to those seen in the controlled mesocosms implies that the physical stress imposed by sediment deposition exerts a major control on these assemblages, as suggested in our previous peatland river surveys (Ramchunder et al., 2012, 2013). Notably, for the three of 11 variables which were different amongst mesocosms vs. rivers (i.e. richness, Ephemeroptera density, FDis), significant declines in response to increasing sediment deposition were observed only in the controlled environment of the mesocosms. Greater nestedness of invertebrate assemblages within mesocosms as sedimentation increased reflects more variance amongst assemblages that originate from a common source pool (Moss Burn) over the short experiment duration, with minimal turnover within treatments potentially related to high experimental control in mesocosm environments. In contrast, turnover was most important for driving beta diversity increases in headwaters, reflecting an exacerbation of patchiness (Winemiller, Flecker, & Hoeinghaus, 2010) due to riverbed sedimentation coupled with longer‐term colonization by a greater number of tolerant taxa from the regional species pool. Together, these findings arguably illustrate the value of riverside mesocosms to control confounding variables effectively, so that effects of the stressor of interest can be identified on these response variables more clearly than in field surveys.

Evidence that riverside mesocosm stressor experiments can mimic invertebrate community responses being seen in peatland headwater networks was particularly strong for taxonomic responses, whereas trait‐based responses were only detected in the controlled environs of the mesocosm. Our study illustrates that more complete understanding of the mechanistic basis of invertebrate biodiversity responses to organic sedimentation requires refinement of trait databases, similar to studies focused primarily on inorganic sediments (Wilkes et al., 2017). While we focused on invertebrate communities in our experimental study spanning week‐ to month‐long sediment deposition events, previous work in the UK uplands showed that even short‐term (1–2 day) pulses of sediment cause effects throughout the whole aquatic ecosystem, including water quality, invertebrate drift, invertebrate community structure and ecosystem metabolism (Aspray et al., 2017). Such pulses are likely to occur particularly with rainfall after summer desiccation events and after needle ice weathering in winter/spring (Li et al., 2016; Li, Holden, & Grayson, 2018a). The former are likely to be key drivers of enhanced peat erosion under future climate change (Li, Irvine, et al., 2017b). The negative effects of organic sediment deposition spanning multiple levels of ecological organization can also be expected across peatland river networks where land management enhances soil erosion and transport to watercourses. However, land managers can limit the erosion and delivery of organic sediments to aquatic systems in intensively managed peatlands with approaches such as ditch and gully blocking, and creating pool systems to trap sediment and reduce rates of overland flow (Holden, Gascoign, & Bosanko, 2006; Ramchunder et al., 2012), preventing the exposure of peat by removing vegetation, while reseeding and planting bare areas (Shuttleworth, Evans, Hutchinson, & Rothwell, 2015), and leaving buffer zones (O’Driscoll et al., 2014) or creating storm‐water retention ponds (Marttila & Kløve, 2010) when harvesting forests.

## 5 SUMMARY

Peatlands are major stores of organic carbon throughout temperate and sub‐Arctic regions, but in addition to land‐use drivers of erosion, widespread, but spatially variable increases in erosion of blanket peat have been predicted due to climate change by 2100 in models driven by several different global climate models (Li, Holden, et al., 2017a). Forecasts of climate change impacts on peatland erosion in the United Kingdom have also shown that large increases in peat erosion are likely by the end of the 21st century, even if land management was optimized for peatland protection (Li, Irvine, et al., 2017b). Effects of increased evapotranspiration and enhanced desiccation of peat at both high and low latitudes are generally expected to drive enhanced peat erosion. Warming of currently frozen Arctic permafrost soils, many of which contain major peat deposits, is also expected to lead to eventual desiccation and erosion (Swindles et al., 2015). Linking our findings of strong responses amongst river invertebrate communities to sedimentation with predicted future changes in blanket, permafrost bog and fen peatlands, leads to the conclusion that climate change can be expected to drive widespread degradation of peatland river ecosystems across the Northern Hemisphere.

## CONFLICT OF INTEREST

The authors declare no conflict of interests.
