# Supplementary material for: Sediment deposition from eroding peatlands alters headwater invertebrate biodiversity
Source: Glob Chang Biol. 2018 Dec 1;25(2):602–19. doi: 10.1111/gcb.14516 (PMC7380017; doi:10.1111/gcb.14516)
Supplement: Supplementary file 1 [file GCB-25-602-s001.docx]

**Supplementary information**

**
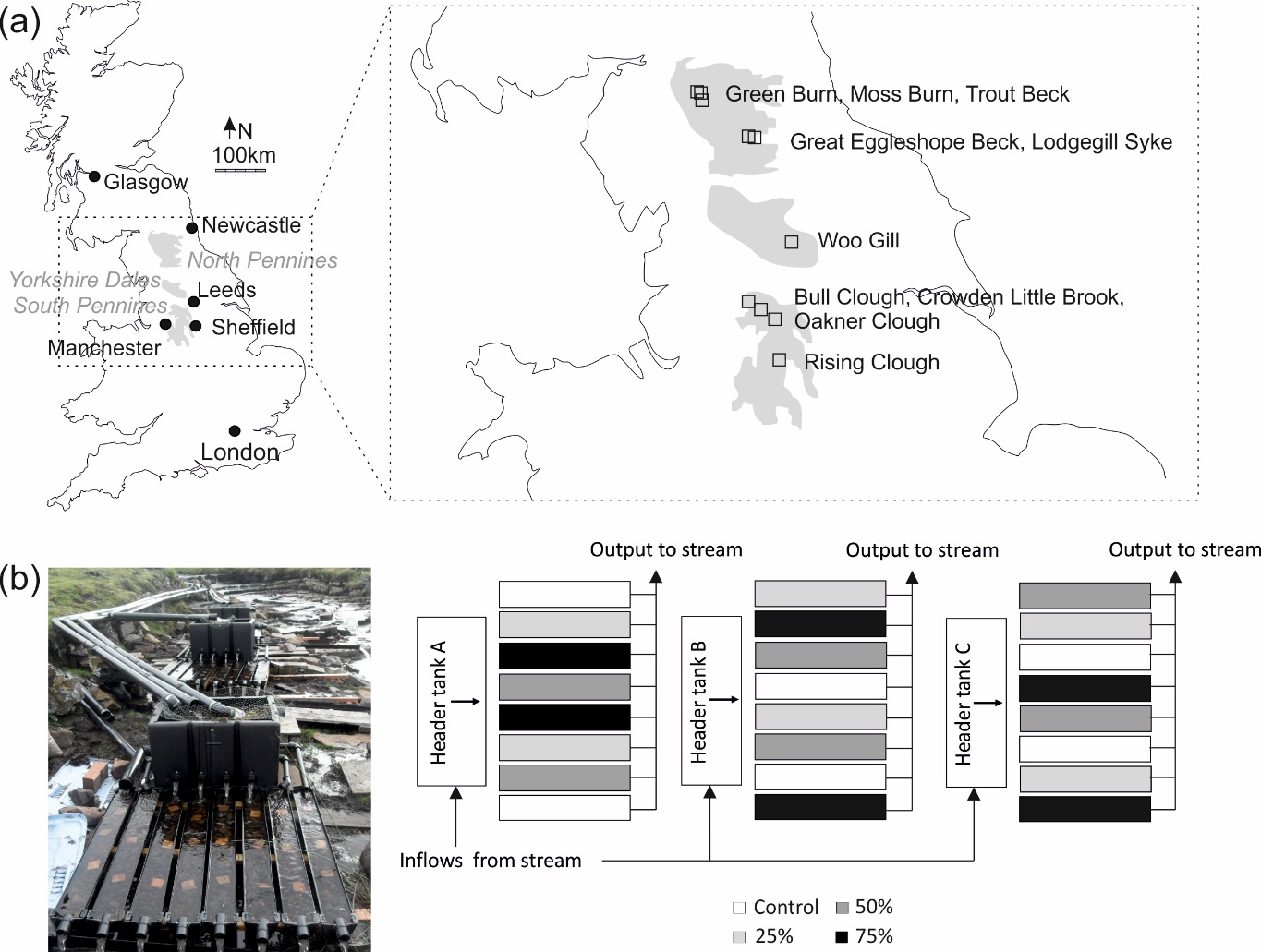
**

**Supplementary Figure 1.** Experimental set up: (a) map of Pennine region headwater survey sites, and (b) mesocosm arrays showing randomised position of treatments

Supplementary Figure 2. Relative abundance of macroinvertebrate taxonomic groups in mesocosm experiment treatments and peatland headwater surveys

Supplementary Table 1. Mean physicochemical parameters for mesocosm treatments and Moss Burn, with ANOVA summary results included from between-treatment comparisons each week using fully replicated data.

|  | **pH** | | | | **DO (mg L^-1^)** | | | | **Temperature (^o^C)** | | | | **EC (µS cm^-1^)** | | | |
| --- | --- | --- | --- | --- | --- | --- | --- | --- | --- | --- | --- | --- | --- | --- | --- | --- |
|  | **Week** | | | | **Week** | | | | **Week** | | | | **Week** | | | |
|  | 1 | 2 | 3 | 4 | 1 | 2 | 3 | 4 | 1 | 2 | 3 | 4 | 1 | 2 | 3 | 4 |
| Moss Burn | 6.2 | 7.4 | 7.5 | 7.1 | 12.2 | 10.1 | 11.3 | 10.8 | 5.4 | 5.7 | 6.9 | 7.1 | 15.2 | 37.7 | 63.9 | 59.8 |
| Control | 6.2 | 7.2 | 7.4 | 7.1 | 12.5 | 10.1 | 11.2 | 10.8 | 5.4 | 5.9 | 6.8 | 7.1 | 15.2 | 37.8 | 63.9 | 59.4 |
| 2.5 g m^-2^ | 6.2 | 7.2 | 7.4 | 7.2 | 12.5 | 10.1 | 11.3 | 10.9 | 5.4 | 5.9 | 6.8 | 7.0 | 15.2 | 37.8 | 63.8 | 59.7 |
| 5 g m^-2^ | 6.2 | 7.2 | 7.4 | 7.2 | 12.4 | 10.0 | 11.2 | 10.8 | 5.4 | 5.8 | 6.8 | 7.0 | 15.3 | 37.8 | 63.8 | 59.8 |
| 7.5 g m^-2^ | 6.2 | 7.2 | 7.4 | 7.2 | 12.5 | 10.0 | 11.2 | 10.9 | 5.4 | 5.9 | 6.8 | 7.0 | 15.2 | 37.6 | 63.8 | 59.6 |
| *p value* | 0.8 | 0.6 | 0.3 | 0.5 | 0.6 | 0.5 | 0.8 | 0.8 | 0.8 | 0.6 | 0.3 | 0.5 | 0.2 | 0.4 | 0.3 | 0.1 |

**Supplementary Table 2.** Macroinvertebrate traits used from Tachet et al. (2010).

| **Category** | **Mode** | **Short name** |
| --- | --- | --- |
| Maximum length (mm) | <2.5 | VSmall |
|  | 2.5-5 | Small |
|  | 5-10 | SmallMed |
|  | 10-20 | Med |
|  | 20-40 | MedLarge |
|  | 40-80 | Large |
|  | >80 | VLarge |
| Life cycle duration (years) | ≤1 | Ephem |
|  | >1 | Perren |
| Voltinism (generations per year) | <1 | Semivoltine |
|  | 1 | Univoltine |
|  | >1 | Multivoltine |
| Aquatic stages | Egg | Egg |
|  | Larva | Larva |
|  | Nymph | Nymph |
|  | Adult | Adult |
| Reproduction | Ovoviviparous and care for young | Ovoviviparity |
|  | Free single eggs | IsolatedEggsFree |
|  | Fixed single eggs | IsolatedEggsCemented |
|  | Cemented or fixed clutches | ClutchesFixed |
|  | Free clutches | ClutchesFree |
|  | Endophytic clutches | ClutchesVeg |
|  | Terrestrial clutches | ClutchesTerr |
|  | Asexual reproduction | Asexual |
| Dispersal | Water passive | AquaticPassive |
|  | Water active | AquaticActive |
|  | Areial passive | AerialPassive |
|  | Aerial active | AerialActive |
| Resistance forms | Eggs, statoblasts | EggsStatoblasts |
|  | Cocoons | Cocoons |
|  | Protection against dessication | Housing |
|  | Diapause/dormancy | DiapauseDormancy |
|  | None | NoResistance |
| Type of food | Fine sediment and microrganisms | Microorganisms |
|  | Detritus <1mm | FineDetritus |
|  | Plant detritus >1mm | DeadPlant |
|  | Living microphytes | Microphytes |
|  | Living macrophytes | Macrophytes |
|  | Dead animals >1mm | DeadAnimal |
|  | Living microinvertebrates | Microinvs |
|  | Living macroinvertebrates | Macroinvs |
|  | Vertebrates | Vertebrates |
| Feeding mode | Absorber | Absorber |
|  | Collector-gatherer | DepositFeeder |
|  | Shredder | Shredder |
|  | Scraper | Scraper |
|  | Filterer | Filterer |
|  | Piercer | Piercer |
|  | Predator | Predator |
|  | Parasite | Parasite |
| Respiration | Tegument | Tegument |
|  | Gills | Gill |
|  | Plastron | Plastron |
|  | Spiracle | Spiracle |
|  | Hydrostatic vesicle | Hydrostatic |
| Mode of locomotion and relationship to substrate | Flight | Flier |
|  | Surface swimmers | SurfaceSwimmer |
|  | Open water swimmer | OpenWaterSwimmer |
|  | Crawling | Crawler |
|  | Burrowing | Burrower |
|  | Within interstices | Interstitial |
|  | Attached (temporary) | AttachedTemp |
|  | Attached (permanent) | AttachedPerm |

**Supplementary Table 3.** Linear mixed-effect model summaries for all response variables examined in the mesocosm experiments. Significant p values highlighted in bold.

| **Response variable** | **Model** | | | |
| --- | --- | --- | --- | --- |
|  | **Response ~ FPOM** | **Response ~ FPOM + (1\|block)** | **Response ~ FPOM + (1\|replicate)** | **Response ~ FPOM + (1\|block/replicate)** |
| Temperature | t=1.41; p=0.17;  R^2^=0.08; AIC=-76.33 | t=1.44; p=0.17; R^2^_m_=0.08; AIC=-57.29 | t=1.41; p=0.18; R^2^_m_=0.08; AIC=-57.14 | t=1.44; p=0.17; R^2^_m_=0.08; AIC=-55.29 |
| pH | t=0.33; p=0.75;  R^2^ =0.005; AIC=-25.83 | t=0.79; p=0.44; R^2^_m_=0.004; AIC=-41.60 | t=1.03; p=0.32; R^2^_m_=0.004; AIC=-42.47 | t=1.03; p=0.33; R^2^_m_=0.004; AIC=-44.73 |
| EC | t=-1.2; p=0.24;  R^2^=0.06; AIC=-17.35 | t=-1.22; p=0.23; R^2^_m_=0.058; AIC=-3.23 | t=-1.34; p=0.20; R^2^_m_=0.058; AIC=-4.26 | t=1.34; p=0.20; R^2^_m_=0.058; AIC=-2.26 |
| SSC | t=-0.64; p=0.52;  R^2^=0.02; AIC=70.91 | t=-0.64; p=0.53; R^2^_m_=0.018; AIC=77.83 | t=-0.64; p=0.53; R^2^_m_=0.018; AIC=77.83 | t=-0.64; p=0.53; R^2^_m_=0.018; AIC=79.83 |
| DOC | t=-0.39; p=0.70;  R^2^=0.007; AIC=155.80 | t=-0.43; p=0.67; R^2^_m_=0.006; AIC=153.62 | t=-0.45; p=0.63; R^2^_m_=0.006; AIC=153.63 | t=-0.45; p=0.65; R^2^_m_=0.006; AIC=155.22 |
| TON | t=2.92; **p=0.008**;  R^2^=0.28; AIC=-103.80 | t=2.92; **p=0.008**; R^2^_m_=0.27; AIC=-82.33 | t=2.95; **p=0.009**; R^2^_m_=0.27; AIC=-82.34 | t=2.95; **p=0.009**; R^2^_m_=0.27; AIC=-80.34 |
| Density | t=-3.00; **p=0.007**;  R^2^=0.29; AIC=353.80 | t=-3.09; **p=0.006**; R^2^_m_=0.28; AIC=336.88 | t=-3.32; **p=0.004**; R^2^_m_=0.28; AIC=336.11 | t=-3.32; **p=0.004**; R^2^_m_=0.28; AIC=338.11 |
| Taxonomic richness | t=-3.90; **p=0.0008**;  R^2^=0.41; AIC=126.18 | t=-3.89; **p=0.0009**; R^2^_m_=0.40; AIC=128.49 | t=-4.63; **p=0.0002**; R^2^_m_=0.39; AIC=126.05 | t=-4.63; **p=0.0002**; R^2^_m_=0.39; AIC=128.05 |
| Beta diversity (Sorensen) | t=5.74; **p<0.00001**;  R^2^=0.36; AIC=-76.41 | t=6.31; **p<0.00001**; R^2^_m_=0.34; AIC=-64.02 | t=6.16; **p<0.00001**; R^2^_m_=0.36; AIC=-60.86 | t=6.31; **p<0.00001**; R^2^_m_=0.34; AIC=-62.02 |
| Beta diversity (Turnover) | t=1.19; p=0.24;  R^2^=0.02; AIC=5.09 | t=1.19; p=0.24; R^2^_m_=0.02; AIC=19.33 | t=1.19; p=0.24; R^2^_m_=0.02; AIC=19.33 | t=1.19; p=0.24; R^2^_m_=0.02; AIC=21.33 |
| Beta diversity (Nestedness) | t=2.15; **p=0.036**;  R^2^=0.07; AIC=-22.16 | t=2.15; **p=0.036**; R^2^_m_=0.07; AIC=-7.01 | t=2.15; **p=0.037**; R^2^_m_=0.07; AIC=-7.01 | t=2.15; **p=0.037**; R^2^_m_=0.07; AIC=-5.01 |
| Chironomidae | t=-1.62; p=0.12;  R^2^=0.11; AIC=334.74 | t=-1.81; p=0.08; R^2^_m_=0.10; AIC=317.48 | t=-1.77; p=0.09; R^2^_m_=0.10; AIC=318.87 | t=-1.82; p=0.09; R^2^_m_=0.10; AIC=319.48 |
| Ephemeroptera | t=-3.10; **p=0.005**;  R^2^=0.30; AIC=231.86 | t=-3.10; **p=0.006**; R^2^_m_=0.30; AIC=225.36 | t=-3.10; **p=0.007**; R^2^_m_=0.30; AIC=225.37 | t=-3.10; **p=0.007**; R^2^_m_=0.30; AIC=227.37 |
| Plecoptera | t=-4.02; **p=0.0006**;  R^2^=0.42; AIC=309.13 | t=-4.02; **p=0.0007**; R^2^_m_=0.41; AIC=296.19 | t=-4.54; **p=0.0003**; R^2^_m_=0.41; AIC=294.82 | t=-4.54; **p=0.0003**; R^2^_m_=0.41; AIC=296.82 |
| Coleoptera | t=-1.87; p=0.07;  R^2^=0.14; AIC=185.90 | t=-1.87; p=0.08; R^2^_m_=0.13; AIC=183.23 | t=-1.87; p=0.08; R^2^_m_=0.13; AIC=185.23 | t=-1.87; p=0.08; R^2^_m_=0.13; AIC=185.23 |
| *L. inermis* | t=-4.08; **p=0.0005**;  R^2^=0.43; AIC=185.85 | t=-4.08; **p=0.0006**; R^2^_m_=0.42; AIC=183.19 | t=-4.51; **p=0.0003**; R^2^_m_=0.42; AIC=182.21 | t=-4.51; **p=0.0003**; R^2^_m_=0.42; AIC=184.21 |
| Shredder | t=-4.15; **p=0.0004**;  R^2^=0.44; AIC=255.44 | t=-4.15; **p=0.0005**; R^2^_m_=0.43; AIC=246.98 | t=-4.77; **p=0.0002**; R^2^_m_=0.42; AIC=245.25 | t=-4.77; **p=0.0002**; R^2^_m_=0.42; AIC=247.25 |
| FRic | t=-3.04; p=0.76;  R^2^=0.005; AIC=0.84 | t=-0.30; p=0.76; R^2^_m_=0.004; AIC=13.36 | t=-0.53; p=0.60; R^2^_m_=0.01; AIC=12.65 | t=-0.53; p=0.60; R^2^_m_=0.01; AIC=14.65 |
| FDis | t=-2.02; p=0.056;  R^2^=0.16; AIC=92.53 | t=-2.17; **p=0.042**; R^2^_m_=0.14; AIC=96.35 | t=-2.30; **p=0.034**; R^2^_m_=0.15; AIC=96.02 | t=-2.30; **p=0.034**; R^2^_m_=0.14; AIC=97.83 |

**Supplementary Table 4.**  Tukey post-hoc test significance (p) results from linear mixed-effect models for all response variables examined in the mesocosm experiments. Significant p values highlighted in bold.

|  | **Density**  **(inds. m^-2^)** | **Taxonomic Richness** | **Beta diversity** | **Chironomidae (inds. m^-2^)** | **Ephemeroptera**  **(inds. m^-2^)** | **Plecoptera**  **(inds. m^-2^)** | **Coleoptera**  **(inds. m^-2^)** | ***L. inermis***  **(inds. m^-2^)** | **Shredder (inds. m^-2^)** | **FRic** | **FDis** |
| --- | --- | --- | --- | --- | --- | --- | --- | --- | --- | --- | --- |
| Control: 2.5g m^-2^ | 0.99 | 0.44 | 0.99 | 0.99 | 1.00 | 0.72 | 0.05 | 0.90 | 0.61 | 0.92 | 0.99 |
| Control: 5.0g m^-2^ | 0.20 | **0.003** | 0.08 | 0.88 | 0.49 | **0.004** | 0.19 | **0.01** | **0.003** | 0.82 | 0.74 |
| Control: 7.5g m^-2^ | **0.03** | **<0.001** | **<0.001** | 0.43 | **0.03** | **<0.001** | 0.11 | **<0.001** | **<0.001** | 1.00 | 0.16 |
| 2.5g m^-2^: 5g m^-2^ | 0.29 | 0.22 | 0.17 | 0.74 | 0.49 | 0.09 | 0.94 | 0.08 | 0.10 | 0.99 | 0.90 |
| 2.5g m^-2^: 7.5g m^-2^ | **0.047** | 0.05 | **<0.001** | 0.28 | **0.03** | **0.02** | 0.99 | **0.009** | **0.02** | 0.99 | 0.30 |
| 5.0g m^-2^: 7.5g m^-2^ | 0.84 | 0.92 | **0.01** | 0.86 | 0.55 | 0.96 | 0.99 | 0.87 | 0.94 | 0.95 | 0.72 |

**Supplementary Table 5.**  Results of the fourth-corner analysis testing for significant correlations between mesocosm experimental treatment and single traits, reporting values of Pearson’s r. Traits with unadjusted p≤0.10 were considered most strongly linked to environment for the purposes of illustrating results of the RLQ analysis (Figure 4 & 5). See Supplementary Table 2 for trait codes.

| **Trait** | **Standardised value** | ***p*** | ***p_adj_*** |
| --- | --- | --- | --- |
| VSmall | 0.96 | 0.35 | 1 |
| Small | 1.71 | 0.09 | 1 |
| SmallMed | -0.50 | 0.63 | 1 |
| Med | -0.29 | 0.80 | 1 |
| MedLarge | 1.65 | 0.10 | 1 |
| Large | 2.19 | 0.03 | 1 |
| VLarge | 0.96 | 0.35 | 1 |
| Ephem | 0.71 | 0.50 | 1 |
| Perren | -1.63 | 0.11 | 1 |
| Semivoltine | -2.17 | 0.03 | 1 |
| Univoltine | -1.80 | 0.08 | 1 |
| Multivoltine | 1.86 | 0.07 | 1 |
| Egg | -1.58 | 0.12 | 1 |
| Larva | -1.87 | 0.07 | 1 |
| Nymph | 1.62 | 0.12 | 1 |
| Adult | 0.94 | 0.34 | 1 |
| Ovoviviparity | 0.88 | 0.41 | 1 |
| IsolatedEggsFree | 1.65 | 0.11 | 1 |
| IsolatedEggsCemented | -1.89 | 0.06 | 1 |
| ClutchesFixed | 1.16 | 0.25 | 1 |
| ClutchesFree | 1.42 | 0.16 | 1 |
| ClutchesVeg | -0.10 | 1.00 | 1 |
| ClutchesTerr | 1.75 | 0.09 | 1 |
| Asexual | 0.96 | 0.35 | 1 |
| AquaticPassive | -1.08 | 0.29 | 1 |
| AquaticActive | -1.88 | 0.06 | 1 |
| AerialPassive | 1.61 | 0.12 | 1 |
| AerialActive | -1.26 | 0.22 | 1 |
| EggsStatoblasts | -1.65 | 0.10 | 1 |
| Cocoons | 1.26 | 0.22 | 1 |
| Housing | NA | 1.00 | 1 |
| DiapauseDormancy | -0.01 | 0.99 | 1 |
| NoResistance | -0.78 | 0.47 | 1 |
| Tegument | -0.33 | 0.75 | 1 |
| Gill | 1.56 | 0.11 | 1 |
| Plastron | -0.34 | 0.77 | 1 |
| Spiracle | 1.47 | 0.14 | 1 |
| Hydrostatic | 1.89 | 0.04 | 1 |
| Flier | -0.43 | 0.69 | 1 |
| SurfaceSwimmer | 1.89 | 0.04 | 1 |
| OpenWaterSwimmer | 0.98 | 0.37 | 1 |
| Crawler | -1.96 | 0.06 | 1 |
| Burrower | -0.86 | 0.40 | 1 |
| Interstitial | 1.17 | 0.26 | 1 |
| AttachedTemp | 1.72 | 0.09 | 1 |
| AttachedPerm | 0.96 | 0.35 | 1 |
| Microorganisms | -0.70 | 0.49 | 1 |
| FineDetritus | 2.00 | 0.05 | 1 |
| DeadPlant | -1.10 | 0.28 | 1 |
| Microphytes | 1.39 | 0.18 | 1 |
| Macrophytes | -1.29 | 0.21 | 1 |
| DeadAnimal | -1.69 | 0.10 | 1 |
| Microinvs | 1.34 | 0.19 | 1 |
| Macroinvs | -1.79 | 0.07 | 1 |
| Vertebrates | -0.49 | 0.67 | 1 |
| Absorber | 0.96 | 0.35 | 1 |
| DepositFeeder | 0.84 | 0.41 | 1 |
| Shredder | -1.77 | 0.08 | 1 |
| Scraper | 1.48 | 0.15 | 1 |
| Filterer | 1.79 | 0.08 | 1 |
| Piercer | -0.19 | 0.85 | 1 |
| Predator | -0.42 | 0.69 | 1 |
| Parasite | 1.62 | 0.12 | 1 |

**Supplementary Table 6.** Taxon list for mesocosm and headwater river surveys. Abundances are means (St.Dev) across all replicates collected in the mesocosms and headwater streams

| **Taxon** | **Mesocosm channels** | **Headwater rivers** |
| --- | --- | --- |
| Ameletus inopinatus | 11.7 (18.3) | 3.6 (11.9) |
| Amphinemura sp. | 2.1 (5.1) |  |
| Amphinemura standfussi | 1.3 (3.4) | 40.8 (114.9) |
| Amphinemura sulcicollis | 6.3 (13.5) | 0.4 (2.8) |
| Baetis rhodani | 2.5 (6.1) |  |
| Baetis sp. | 8.8 (13.0) | 1.6 (5.5) |
| Baetis sp.2 | 3.3 (6.4) |  |
| Brachyptera risi | 0.4 (2.0) | 1.6 (6.8) |
| Capnia bifrons |  | 0.4 (2.8) |
| Capnia sp. | 1.7 (3.8) |  |
| Capnia vidua |  | 2.4 (10.4) |
| Centroptilum | 0.4 (2.0) |  |
| Chaetopteryx villosa |  | 1.2 (6.3) |
| Chironomidae | 290.4 (246.7) | 137.6 (276.8) |
| Chloroperla torrentium | 7.9 (15) |  |
| Collembola | 1.3 (4.5) | 8.4 (19.4) |
| Culicoidea | 1.7 (4.8) |  |
| Dicranota sp1 | 2.5 (4.4) | 2.8 (14.6) |
| Dicranota sp2 |  | 4.8 (13.1) |
| Diura bicaudata |  | 1.2 (6.3) |
| Dolichopodidae | 0.4 (2.0) |  |
| Drusus annulatus |  | 2.4 (8.7) |
| Dytiscidae sp. |  | 0.4 (2.8) |
| Ecdyonurus torrentis | 2.9 (6.9) |  |
| Electrogena lateralis | 5.4 (6.6) | 0.4 (2.8) |
| Elmis aenea | 0.4 (2.0) | 0.4 (2.8) |
| Empididae | 1.7 (3.8) |  |
| Esolus parallelepipedus | 0.8 (2.8) |  |
| Gammarus pulex | 3.3 (8.2) |  |
| Helophorus brevipalpis | 1.3 (3.4) |  |
| Heptageniidae sp. | 0.4 (2.0) |  |
| Hydracarina |  | 0.8 (5.7) |
| Hydraena gracilis | 0.4 (2.0) |  |
| Hydraena sp. | 1.3 (4.5) |  |
| Hygrotus (Coelambus) sp. | 1.7 (3.8) |  |
| Isoperla grammatica | 7.5 (13.9) |  |
| Leptophlebia marginata |  | 1.6 (6.8) |
| Leuctra fusca | 0.8 (2.8) |  |
| Leuctra hippopus | 5.4 (11.8) |  |
| Leuctra inermis | 182.9 (138.9) | 298.8 (410.2) |
| Leuctridae sp. | 2.1 (7.2) | 1.6 (8.9) |
| Limnephilidae sp. |  | 13.6 (60.4) |
| Limnius volckmari | 0.4 (2.0) |  |
| Nemoura erratica | 0.4 (2.0) | 5.2 (31.2) |
| Nemoura sp. | 4.2 (8.3) | 92.0 (156.6) |
| Nemouridae sp. | 0.4 (2.0) |  |
| Normandia nitens | 0.4 (2.0) |  |
| Oligochaeta | 8.8 (11.9) | 8.8 (23.6) |
| Ostracoda |  | 0.8 (4.0) |
| Perlodidae sp. | 0.8 (2.8) | 0.4 (2.8) |
| Platambus maculatus | 1.3 (4.5) |  |
| Plectrocnemia geniculata | | 0.8 (5.7) |
| Polycentropodidae sp. | 0.8 (2.8) | 0.4 (2.8) |
| Polycentropus flavomaculatus | 0.8 (2.8) | 4.4 (16.8) |
| Protonemura |  | 0.8 (4.0) |
| Rhithrogena semicolorata | 4.2 (9.3) |  |
| Riolus cupreus | 0.4 (2.0) |  |
| Sialis sp. | 2.1 (5.9) |  |
| Simuliidae | 1.3 (3.4) | 18 (31.9) |
| Tipuildae | 0.4 (2.0) | 0.8 (5.7) |
